# Polystyrene Nanoplastics Drive β‐Cell Dedifferentiation Through Dendritic Cell‐Intrinsic MHC‐I‐Dependent Inflammatory Crosstalk

**DOI:** 10.1002/advs.77096

**Published:** 2026-08-17

**Authors:** Conghui Qiao, Yuqing Song, Fang Yang, Menghui Guo, Libin Jiang, Xiaoyue Quan, Wei Wei, Xinyang Wang, Tianshu Han, Mingyuan Liu, Wenbo Jiang

**Affiliations:** ^1^ Key Laboratory of Precision Nutrition and Health Ministry of Education Department of Nutrition and Food Hygiene the National Key Discipline School of Public Health Harbin Medical University Harbin People's Republic of China; ^2^ Department of Neurology The First Affiliated Hospital of Anhui Medical University Anhui Medical University Hefei People's Republic of China; ^3^ Department of Toxicology College of Public Health Harbin Medical University Harbin Heilongjiang People's Republic of China; ^4^ Department of Vascular Surgery Beijing Friendship Hospital Capital Medical University Beijing People's Republic of China; ^5^ Department of Cardiology the First Affiliated Hospital of Harbin Medical University Harbin People's Republic of China

**Keywords:** dedifferentiation, immune crosstalk, pancreatic, PS‐NPs, single‐cell sequencing

## Abstract

Environmental nanoplastic exposure is linked to metabolic disorders, yet its impact on pancreatic immune‐endocrine homeostasis and β‐cell identity regulation remains poorly defined. Rats were exposed to polystyrene nanoplastics(PS‐NPs) for 12 weeks under a control or high‐fat diet. Pancreatic injury and phenotypes were assessed biochemically, histologically, and ultrastructurally; single‐nucleus RNA‐seq delineated cell‐specific transcription and intercellular networks. Mechanistic validation used cell co‐cultures with MHC‐I modulation, and translational relevance was assessed in a human exposure cohort. Chronic PS‐NPs exposure exacerbated hyperglycemia and glucose intolerance, and induced pancreatic damage. Single‐nucleus transcriptomics identified β cells and dendritic cells (DCs) as the most responsive populations. PS‐NPs drove β‐cell dedifferentiation, characterized by downregulation of key identity markers (Mafa, Pdx1, Nkx6.1). Concurrently, DCs exhibited a maturation‐like phenotype with robust upregulation of MHC‐I and inflammatory pathways. Ligand‐receptor analysis revealed enhanced proinflammatory crosstalk between DCs and β cells. Functionally, MHC‐I upregulation in DCs activated TLR4/NF‐κB signaling and drove β‐cell dedifferentiation in vitro. Consistently, occupationally exposed individuals showed elevated circulating HLA‐A levels and metabolic abnormalities. These findings identify an MHC‐I‐dependent DCs‐β‐cell inflammatory axis through which PS‐NPs disrupt pancreatic immune‐endocrine homeostasis and promote β‐cell dedifferentiation, revealing mechanisms underlying PS‐NPs‐induced metabolic dysfunction.

## Graphical abstract

Polystyrene nanoplastics disrupt pancreatic immune–endocrine homeostasis by promoting MHC‐I‐associated dendritic cell activation and inflammatory crosstalk with β cells. This immune remodeling drives β‐cell dedifferentiation, impairs insulin secretion, and aggravates metabolic dysfunction, revealing a previously unrecognized immunometabolic mechanism linking environmental nanoplastic exposure to endocrine impairment.

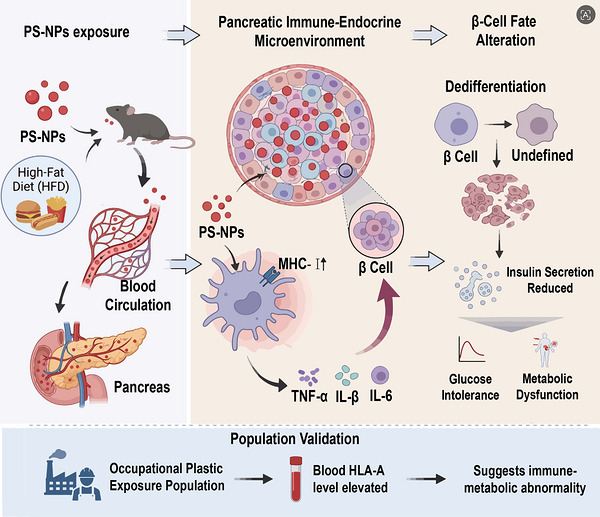

## 1 Introduction

Chronic exposure to PS‐NPs has become an inevitable consequence of modern life, raising increasing concerns about their long‐term metabolic and endocrine consequences [1]. PS‐NPs, the most prevalent and bioactive nanoplastic species, have been shown to disrupt glucose homeostasis and induce systemic metabolic dysfunction [2]. However, current toxicological frameworks largely interpret these effects through the lens of direct cellular toxicity or metabolic stress, potentially overlooking critical immune‐mediated mechanisms within metabolically active organs [3].

The pancreas represents a unique immunometabolic niche in which endocrine cells and resident immune cells are tightly interwoven [4]. Pancreatic β cells maintain glucose homeostasis not only through insulin secretion but also through preservation of a highly differentiated cellular identity [5]. Increasing evidence indicates that loss of β‐cell identity‐rather than irreversible cell death‐is a major driver of β‐cell failure under chronic metabolic stress [6]. Importantly, β‐cell dedifferentiation is a reversible yet highly regulated process, making it a decisive pathological event that determines whether metabolic dysfunction progresses or can be restored [7].

Within this microenvironment, dendritic cells (DCs) play a central role in sensing environmental stress signals and shaping the local inflammatory tone. Beyond their classical immune functions, DCs have been increasingly recognized as active participants in immune–endocrine communication through antigen presentation and cytokine‐mediated signaling [8, 9, 10]. Notably, MHC class I–associated antigen‐presentation pathways have been proposed as potential modulators of immune–endocrine interactions in pancreatic disorders [11]. Emerging evidence suggests that altered MHC‐I signaling in antigen‐presenting cells may influence β‐cell stress responses and differentiation stability, raising the possibility that immune activation could contribute to β‐cell identity perturbation under chronic environmental stress [12, 13].

PS‐NPs are potent activators of innate immune responses, capable of reprogramming antigen‐presenting cells toward proinflammatory states. In the context of the pancreas, such immune activation may fundamentally alter DC‐β‐cell communication, shifting it from physiological immune surveillance to pathological inflammatory crosstalk [14, 15]. Failure to account for this immune‐driven axis may therefore lead to an incomplete or misleading understanding of how PS‐NPs impair pancreatic endocrine function.

In this study, we demonstrate that PS‐NPs drive β‐cell dedifferentiation through a DCs‐intrinsic MHC‐I‐dependent inflammatory crosstalk. By elucidating this immune‐mediated mechanism, our work reframes PS‐NPs‐induced pancreatic dysfunction as a disorder of immune‐regulated cell identity rather than solely direct metabolic toxicity, providing a conceptual framework and potential intervention target with important implications for environmental risk assessment and metabolic disease prevention.

## 2 Method

### 2.1 PS‐NPs Exposure, Animal Model, and Experimental Design

Forty male Sprague‐Dawley (SD) rats (6–8 weeks old) were obtained from Vital River Laboratory Animal Technology Co., Ltd.(Beijing, China). Animals were housed under controlled conditions(12 h light/dark cycle, 18°C–22°C, 40%–50% humidity) with ad libitum access to food and water. After a one‐week acclimatization period, rats were randomly assigned to four groups: control, high‐fat diet(HFD), PS‐NPs group, and HFD+PS‐NPs group. PS‐NPs (80 nm in diameter) were purchased from BaseLine Chromtech Research Centre (Tianjin, China), and particle size, morphology, and surface characteristics were validated as shown in Figure S1. Body weight and food intake were recorded biweekly. At the end of the exposure period, rats were euthanized by intraperitoneal injection of phenobarbital sodium. Blood, pancreas, liver, skeletal muscle, and other tissues were harvested, weighed, and snap‐frozen in liquid nitrogen. All animal procedures were approved by the Medical Ethics Committee of Harbin Medical University and conducted in accordance with the Guide for the Care and Use of Laboratory Animals.

### 2.2 Cell Culture

Human pancreatic β‐cell line 1.1B4 (Cobioer Biosciences Co., Ltd., China) was cultured in RPMI‐1640 medium (Gibco) supplemented with 11.1 mmol/L D‐glucose, 10% fetal bovine serum (FBS; Gibco), 5 µg/mL hydrocortisone (Solarbio), 10 µg/mL insulin (Beyotime), and 1% penicillin–streptomycin. Mouse insulinoma MIN6 cells (Procell, China) were cultured in RPMI‐1640 containing 11.1 mmol/L D‐glucose, 10% FBS, 0.05 mmol/L β‐mercaptoethanol (Sigma), and 1% penicillin–streptomycin. Cells were maintained at 37°C in a humidified incubator with 5% CO_2_. The mouse DC line (DC2.4) was cultured in RPMI‐1640 supplemented with 10% FBS and 1% penicillin–streptomycin under standard conditions.

### 2.3 Histological and Ultrastructural Analyses

Pancreatic tissues were fixed in 2.5% glutaraldehyde followed by post‐fixation in 1% osmium tetroxide. Samples were dehydrated through a graded ethanol and acetone series and embedded in epoxy resin. Ultrathin sections were stained with uranyl acetate and lead citrate and examined using a transmission electron microscope (TEM) to assess mitochondrial morphology, endoplasmic reticulum integrity, and insulin secretory granules. For histopathological examination, paraffin‐embedded pancreatic sections (4 µm) were prepared and stained with hematoxylin and eosin (H&E). Sections were examined under a light microscope by investigators blinded to group allocation.

### 2.4 Enzyme‐Linked Immunosorbent Assay (ELISA)

Pancreatic tissue homogenates and cell culture supernatants were prepared according to standard protocols. Levels of insulin secretion‐related proteins and inflammatory cytokines, including insulin, TNF‐α, and IL‐1β, were measured using commercially available ELISA kits (Jiangsu Meimian Industrial Co., Ltd., China) following the manufacturer's instructions.

### 2.5 Single‐Nucleus RNA Sequencing (snRNA‐seq) and Bioinformatic Analysis

Pancreatic tissues were processed to generate single‐nucleus suspensions, which were used for library preparation with the Chromium Next GEM Single Cell 3′ Reagent Kits v3.1(10×Genomics) on the Chromium Controller. After reverse transcription and cDNA amplification, sequencing libraries were constructed and quality‐controlled using a Qubit 4.0 fluorometer and Agilent 2100 Bioanalyzer. Libraries were sequenced on an Illumina NovaSeq 6000 platform with a minimum depth of 50 000 reads per nucleus (paired‐end 150 bp). Raw sequencing data were processed using Cell Ranger(v3.0.1) with alignment to the mouse reference genome (GRCm38). Gene‐barcode matrices were imported into Seurat(v3.0.2) for downstream analysis. Data normalization, identification of highly variable genes, and dimensionality reduction were performed using standard Seurat workflows.

Batch integration was conducted using the FindIntegrationAnchors and IntegrateData functions. Cell clustering was performed using the Louvain algorithm and visualized by t‐distributed stochastic neighbor embedding(t‐SNE). Cell‐type annotation was achieved using the SingleR package based on canonical marker genes. β cells were further subsetted and reclustered to resolve transcriptional heterogeneity. Trajectory inference and pseudotime analysis were performed using Monocle to reconstruct β‐cell differentiation dynamics. Gene Ontology(GO) and Kyoto Encyclopedia of Genes and Genomes(KEGG) pathway enrichment analyses were conducted using the clusterProfiler package. Cell‐cell communication and ligand‐receptor interaction analyses between DCs and β cells were inferred using CellChat (Figure S2).

### 2.6 Immunofluorescence Staining

Paraffin‐embedded pancreatic sections (5 µm) were deparaffinized, rehydrated, and subjected to antigen retrieval using EDTA buffer (pH 8.0). Sections were blocked with goat serum and incubated with primary antibodies at 37°C for 1 h, followed by Cy3‐conjugated secondary antibodies (1:400, Abcam). Nuclei were counterstained with DAPI, and images were captured using a fluorescence microscope.

### 2.7 Flow Cytometry Analysis

Freshly isolated rat pancreata were enzymatically digested with Collagenase IV (1 mg/mL) at 37°C for 20 min to obtain single‐cell suspensions. Cells were surface‐stained with fluorophore‐conjugated antibodies against immune cell markers for DCs. Data were acquired on an LSRII flow cytometer (BD Biosciences) and analyzed using FlowJo software.

### 2.8 Western Blot Analysis

Total protein was extracted using RIPA buffer supplemented with PMSF. Protein concentration was determined using a BCA assay. Equal amounts of protein were separated by SDS‐PAGE and transferred to PVDF membranes. Membranes were blocked with 5% BSA and incubated overnight at 4°C with primary antibodies against GAPDH, TRAF6, IκB, pIκB, Total NF‐κB, and Nuclear NF‐κB. After incubation with HRP‐conjugated secondary antibodies, bands were visualized by chemiluminescence and quantified using ImageJ software.

### 2.9 DCs‐β‐Cell Interaction Assays and MHC‐I Functional Validation

To investigate the causal role of DCs‐intrinsic MHC class I signaling in regulating β‐cell identity, a non‐contact immune‐endocrine interaction model was established using DCs‐conditioned medium. Mouse DCs(DC2.4) were cultured in RPMI‐1640 medium supplemented with 10% fetal bovine serum and 1% penicillin–streptomycin. For gain‐of‐function experiments, DC2.4 cells were transfected with an H‐2 overexpression plasmid using Lipofectamine 3000, followed by selection with G418 to establish stable overexpression lines. For loss‐of‐function experiments, DC2.4 cells were transfected with short hairpin RNA(shRNA) targeting H‐2 (shH‐2) or a scrambled control using Lipofectamine 3000. After reaching 80%–90% confluence, culture supernatants from control, H‐2‐overexpressing, and shH‐2‐transfected DC2.4 cells were collected, centrifuged to remove cellular debris, and filtered through 0.22 µm membranes. The conditioned media were subsequently applied to human pancreatic β cells (1.1B4) for 24 or 48 h to assess DCs‐mediated paracrine regulation of β‐cell identity. Following treatment, β cells were harvested for analysis of dedifferentiation markers, including Mafa, Pdx1, and Nkx6.1, using quantitative PCR and Western blotting. In parallel, inflammatory cytokine levels (TNF‐α, IL‐1β, IL‐6) in DC‐conditioned media were quantified by ELISA. This non‐contact system was used to model MHC‐I‐dependent inflammatory crosstalk between DCs and β cells.

### 2.10 Human Population Analysis

Data were obtained from the UK Biobank, and detailed information is provided in Table S1. Participants engaged in plastics‐related occupational work were identified based on job codes. Among these individuals, 35 participants with available OLINK proteomic data were included, and 120 matched controls were selected using propensity score matching(1:4 ratio) based on age, sex, BMI, smoking status, and socioeconomic variables. Associations between occupational exposure and circulating HLA‐A level(the human ortholog of RT1‐A1) were evaluated. Participants were further stratified into high and low HLA‐A expression groups to examine differences in glucose metabolism, lipid metabolism, insulin resistance, insulin secretion, and inflammatory markers. All participants provided written informed consent.

### 2.11 Statistical Analysis

Statistical analyses were performed using R software. Data are presented as mean±standard error(SE). Comparisons between two groups were conducted using Student's t‐test or Wilcoxon rank‐sum test, as appropriate. Multiple‐group comparisons were analyzed using one‐way ANOVA followed by post hoc tests. Differential gene expression analysis in single‐nucleus RNA‐seq data was performed using the Wilcoxon rank‐sum test with false discovery rate(FDR) correction. A two‐sided *p* < 0.05 was considered statistically significant.

## 3 Result

### 3.1 PS‐NPs Exacerbate Metabolic Disturbances and Pancreatic Injury in HFD‐fed Rats

**FIGURE 1:**
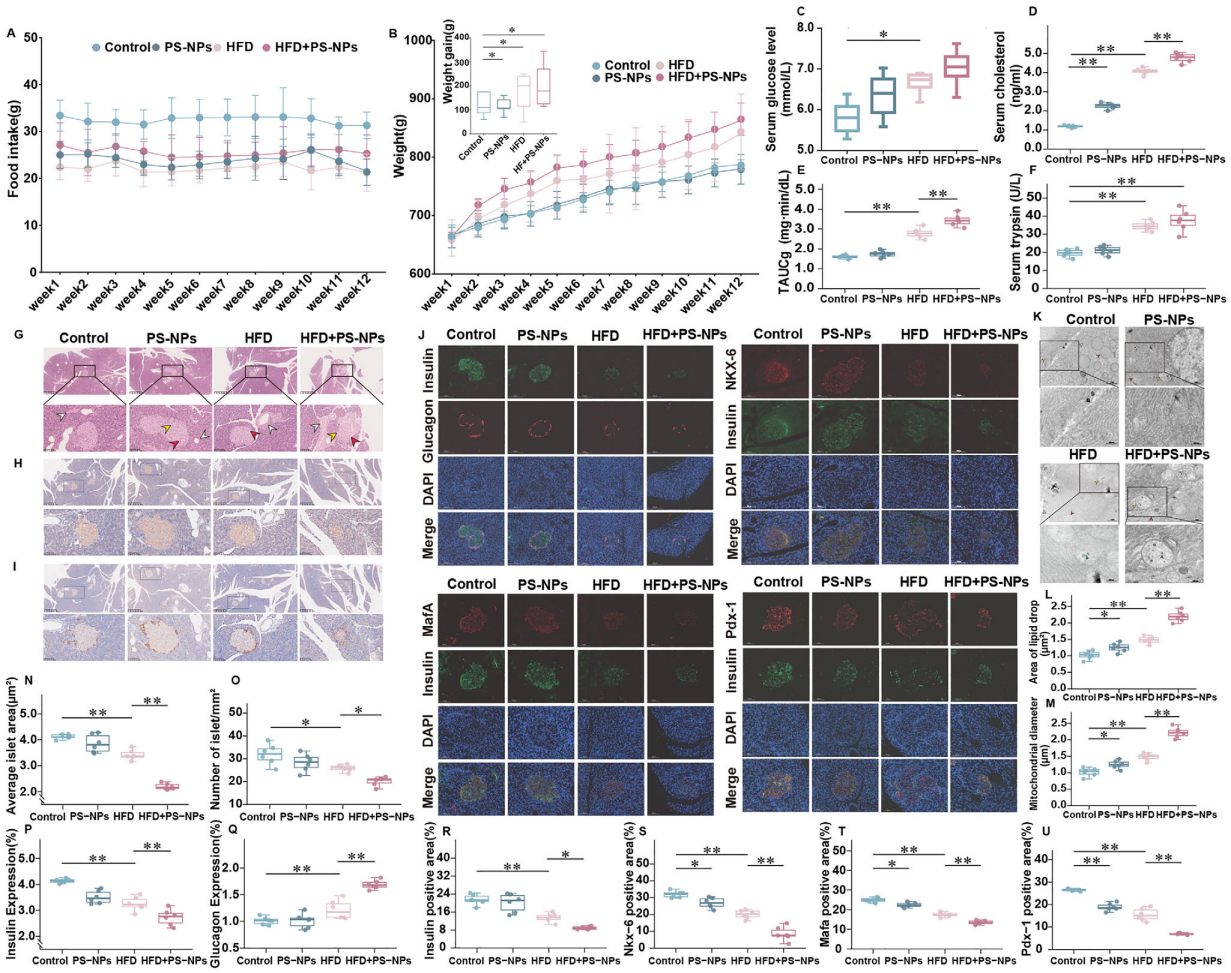
Chronic PS‐NPs exposure exacerbates metabolic dysfunction and pancreatic islet injury in HFD‐fed rats. Rats were treated with PS‐NPs for 12 weeks under either a standard chow diet or a high‐fat diet (HFD). (A) Biweekly food intake. (B) Body weight. (C) Fasting serum glucose. (D) Serum cholesterol. (E) Total area under the curve (TAUCg) for the oral glucose tolerance test (OGTT). (F) Serum trypsin levels. (G) Representative H&E staining of pancreatic sections showing pathological damage. (H–J, P–R) Representative immunofluorescence images and quantitative analysis of (H) insulin (amber‐colored/red) and (I) glucagon (green) positive cells in islets, with (J) and (P–R) showing the proportions relative to total islet cells. (K–M) Ultrastructural analysis by transmission electron microscopy (TEM) showing (K) subcellular structures, with quantitative measurements of (L) lipid drop area and (M) mitochondrial diameter. (N, O) Quantification of (N) average islet area and (O) the number of islets per section. (S–U) Relative mRNA or protein expression levels of β‐cell identity markers: (S) Mafa, (T) Nkx6.1, and (U) Pdx1 in pancreatic tissues. Abbreviations: PS‐NPs, polystyrene nanoplastics; HFD, high‐fat diet; OGTT, oral glucose tolerance test; TAUCg, total area under the curve for glucose; H&E, hematoxylin and eosin; TEM, transmission electron microscope.

To investigate the metabolic impact of PS‐NPs in a context relevant to human lifestyle, we utilized a HFD animal model. The rationale was to determine whether PS‐NPs exposure acts as an environmental ‘second hit’ that exacerbates preexisting metabolic stress, thereby providing a more sensitive platform to observe islet dysfunction. After 12 weeks of exposure, rats receiving PS‐NPs exhibited significantly increased food intake, body weight, serum glucose, serum cholesterol, and OGTT total AUC compared with HFD controls (Figure 1A–E). Serum trypsin levels were unchanged (Figure 1F). Histological examination revealed severe hepatic injury, including hepatocyte necrosis, lipid accumulation, inflammatory infiltration, and fibrosis, while skeletal muscle Glu4 protein expression was markedly reduced (Figure S3), indicating impaired peripheral glucose uptake.

PS‐NPs induced pronounced pancreatic pathology, characterized by disrupted morphology, vacuolization, and reduced islet number and area (3.42 ± 0.20 µm^2^ vs. 2.20 ± 0.12 µm^2^, *p* < 0.01 for average islet area; 25.97 ± 1.47/µm^2^ vs. 20.20 ± 1.96/µm^2^, *p* < 0.01 for number of islet), compared to HFD group (Figure 1G,N,O). Immunohistochemical analysis revealed alterations in the proportions of insulin‐ and glucagon‐positive cells in the HFD+PS‐NPs group(13.38 ± 1.99 vs. 8.8 ± 0.68, *p* < 0.01 for insulin(+) cells/total cells; 1.22 ± 0.18 vs. 1.69 ± 0.08, *p* < 0.01 for glucagon(+) cells/total cells)(Figure 1H–J,P–R). Ultrastructural analysis further revealed cristae disruption, ER expansion, and reduced secretory granules, and area of lipid drop and mitochondrial diameter were remarkably increased in HFD+PS‐NPs rats(1.48 ± 0.11 µm^2^ vs. 2.20 ± 0.16 µm^2^, *p* < 0.01 for area of lipid drop; 1.48 ± 0.11 µm vs. 2.22 ± 0.16 µm, *p* < 0.01 for mitochondrial diameter) (Figure 1K–M). Notably, β‐cell dedifferentiation markers‐including Mafa, Nkx6.1, and Pdx1‐were significantly decreased after PS‐NPs exposure (Figure 1J,S–U), indicating loss of β‐cell identity. While PS‐NPs also induced pathological changes in the chow‐diet group, the combination of HFD and PS‐NPs produced the most severe impairment in glucose tolerance and islet structural integrity. Based on this synergistic effect, we subsequently utilized the HFD‐fed cohorts for high‐resolution single‐nucleus transcriptomic analysis to better resolve the immune‐mediated mechanisms under maximum metabolic stress.

Data information: Data are expressed as Mean±SEM. For animal experiments, *n* = 6 biological replicates (rats) per group for metabolic measurements and histological/TEM analyses. Significant differences are indicated as ^*^ *p* < 0.01, ^**^ *p* < 0.001.

### 3.2 Single‐Nucleus Transcriptomic Landscape Reveals Selective Immune and β‐Cell Reprogramming Induced by PS‐NPs

**FIGURE 2:**
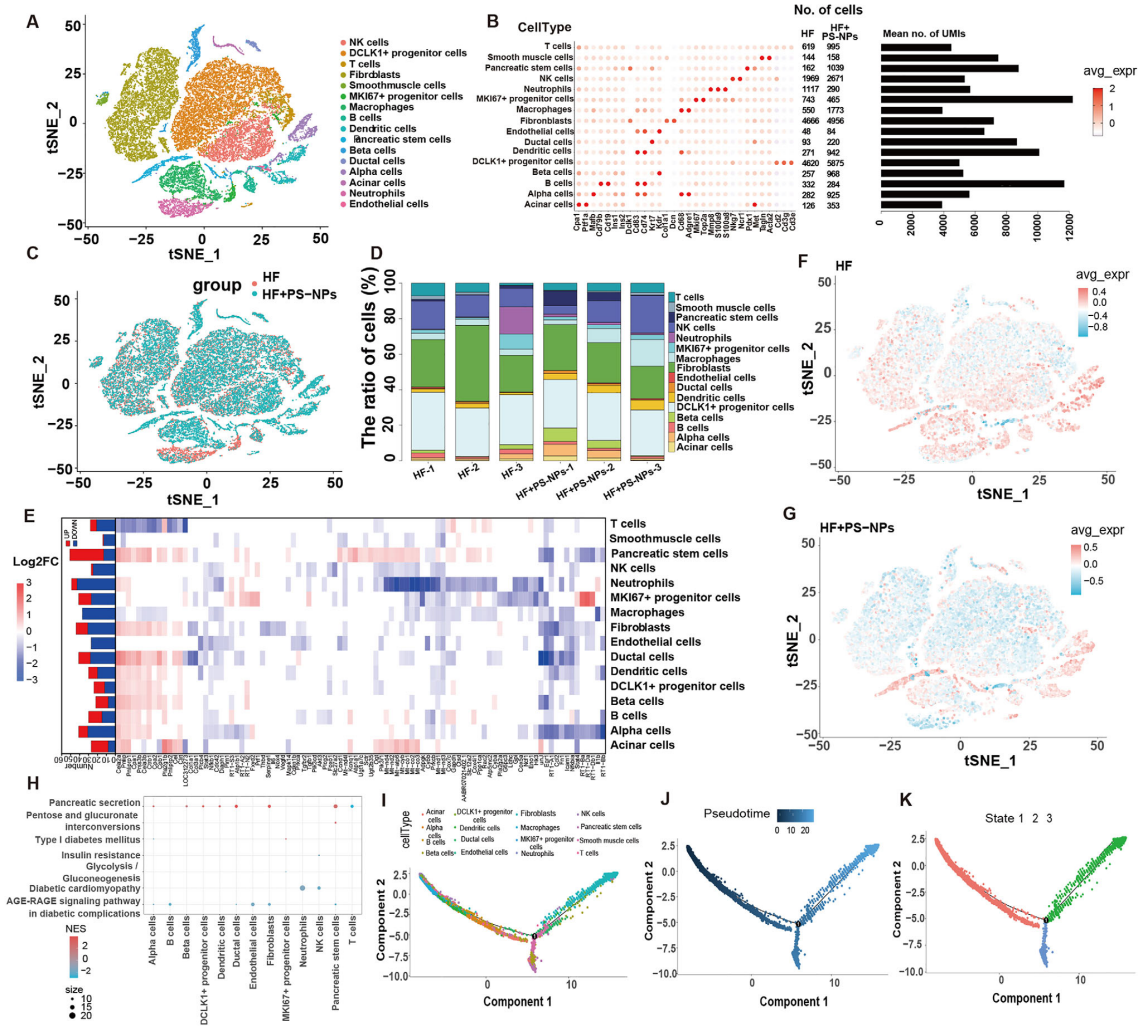
SnRNA‐seq profiling of the rat pancreas after PS‐NPs exposure. Pancreatic tissues from HFD and HFD+PS‐NPs groups were dissociated into single‐nucleus suspensions for 10x Genomics sequencing. (A) t‐SNE plot visualizing 16 transcriptionally distinct cell populations identified via unsupervised clustering. (B) Dot plot showing the expression of canonical marker genes used for cell‐type annotation; dot size indicates the percentage of cells expressing the gene, and color intensity represents average expression. (C, D) t‐SNE plots showing the distribution of cells by experimental group (C) and individual biological replicate (D). (E) Stacked bar plot showing the relative proportion of each cell type across groups. (F, G) Bar plots showing the number of differentially expressed genes (DEGs) total (F) and glucose‐metabolism related (G) for each cluster. (H) Gene Ontology (GO) enrichment analysis of DEGs in major cell types. (I–K) Trajectory inference analysis showing the pseudotime differentiation trajectory of the endocrine compartment. Abbreviations: snRNA‐seq, single‐nucleus RNA sequencing; t‐SNE, t‐distributed stochastic neighbor embedding; HFD, high‐fat diet; PS‐NPs, polystyrene nanoplastics; DEGs, differentially expressed genes; GO, Gene Ontology. Data information: For snRNA‐seq, *n* = 3 biological replicates per group were pooled for library construction. DEG analysis used the Wilcoxon rank‐sum test with FDR correction (*p* < 0.05).

To resolve cell‐type‐specific responses within the heterogeneous pancreas at high resolution, we performed snRNA‐seq. snRNA‐seq of pancreatic tissue from HFD and HFD+PS‐NPs‐exposed rats identified 16 transcriptionally distinct cell populations, including endocrine cells, exocrine cells, immune cells, and stromal components (Figure 2A). Canonical marker expression confirmed robust cell‐type annotation across clusters (Figure 2B). Integration of all samples revealed a largely overlapping global cellular architecture between groups (Figure 2C), while quantitative analysis demonstrated distinct alterations in cell‐type composition following PS‐NPs exposure (Figure 2D). Notably, PS‐NPs exposure was associated with a shift in endocrine cell‐state composition, characterized by an increased proportion of DCs and a reduction in several endocrine and progenitor populations, indicating selective immune remodeling rather than global cellular loss (Figure 2D). Differential expression analysis across major cell types revealed cell‐type–specific transcriptional reprogramming, with pronounced changes observed in β cells and DCs (Figure 2E). Consistent with our snRNA‐seq findings, the flow cytometric analysis (Figure S4) revealed a significant expansion of the DCs population in the HFD+PS‐NPs group. This robust, independent validation confirms that PS‐NPs exposure triggers localized recruitment of DCs to the pancreas.

Feature plots of representative genes further demonstrated group‐specific expression shifts across the pancreatic landscape(Figure 2F,G). Pathway enrichment analysis revealed that DEGs in multiple cell types were significantly associated with pancreatic secretion, insulin resistance, type I diabetes mellitus, and AGE–RAGE signaling pathways, highlighting coordinated metabolic and inflammatory perturbations induced by PS‐NPs exposure(Figure 2H). Trajectory inference analysis uncovered a continuous differentiation axis within the endocrine compartment, with β cells distributed along a pseudotemporal trajectory indicative of progressive identity remodeling (Figure 2I). Stratification by pseudotime and experimental group revealed that PS‐NPs exposure biased β cells toward later pseudotime states(Figure 2J,K), suggesting a shift away from canonical mature β‐cell states, which was further supported by the reduced expression of β‐cell maturity markers described below. Collectively, these results demonstrate that PS‐NPs do not globally disrupt pancreatic cellular composition but instead selectively reshape immune–endocrine interactions, characterized by DCs expansion, metabolic and inflammatory pathway activation, and β‐cell trajectory shifts indicative of dedifferentiation‐related remodeling.

### 3.3 PS‐NPs Reprogram β‐Cell States Toward Immaturity and Stress‐Associated Transcriptional Profiles

**FIGURE 3:**
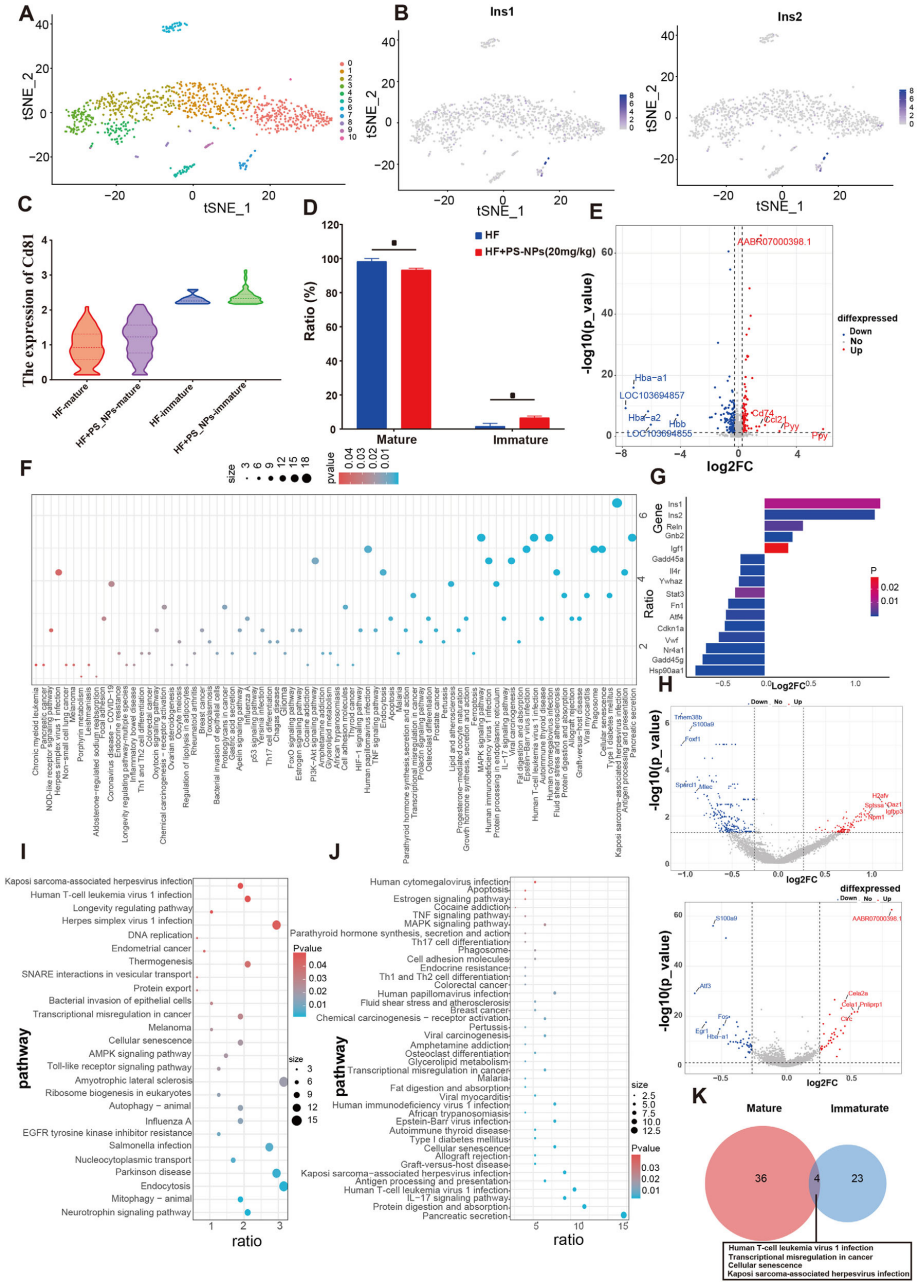
PS‐NPs promote β‐cell state shifts toward immaturity. Detailed subclustering analysis of the β‐cell compartment isolated from the global dataset. (A) t‐SNE visualization of 11 distinct β‐cell subclusters. (B, C, G) Feature plots showing the expression of Ins1, Ins2 (B), and the immaturity marker Cd81 (C, G). (D)Bar plot showing the ratio of mature vs. immature β cells following PS‐NPs exposure. (E) Volcano plot showing 141 upregulated and 141 downregulated DEGs in β cells. (F, I–K) KEGG pathway enrichment analysis for total β‐cell DEGs (F), mature‐specific (I), immature‐specific (J), and shared mutual pathways (K). Abbreviations: t‐SNE, t‐distributed stochastic neighbor embedding; DEGs, differentially expressed genes; KEGG, Kyoto Encyclopedia of Genes and Genomes. Data information: *n* = 3 biological replicates per group. Statistical significance for subclustering was determined using the Louvain algorithm.

Subclustering analysis of β cells resolved 11 transcriptionally distinct β‐cell states, revealing pronounced cellular heterogeneity within the β‐cell compartment (Figure 3A). Canonical insulin markers Ins1 and Ins2 were broadly expressed across β‐cell clusters, confirming accurate β‐cell identification (Figure 3B). Comparative analysis between groups demonstrated that PS‐NPs exposure significantly altered β‐cell state composition, characterized by a reduced proportion of mature β cells and a concomitant expansion of immature β‐cell populations (Figure 3C,D). Differential expression analysis identified extensive transcriptional remodeling in β cells following PS‐NPs exposure, with 141 upregulated and 141 downregulated DEGs (Figure 3E). Notably, genes associated with β‐cell immaturity and stress responses, including Cd81, were significantly increased, whereas markers linked to β‐cell maturity were diminished. Consistently, PS‐NPs exposure markedly elevated Cd81 expression, particularly within immature β‐cell subsets (Figure 3G).

To further validate the β‐cell state transition suggested by snRNA‐seq, we performed qPCR analysis in isolated pancreatic islets. PS‐NPs exposure significantly reduced the expression of key β‐cell identity genes (Pdx1 and Mafa), while increasing the expression of dedifferentiation‐associated markers (Aldh1a3 and Sox9). In parallel, the cellular stress markers Atf4 and Ddit3 were markedly upregulated. In contrast, classical senescence‐associated genes, including Cdkn1a and Cdkn2a, showed minimal changes between groups (Figure S5). These findings provide independent evidence that PS‐NPs exposure induces β‐cell identity loss accompanied by stress‐associated remodeling and a dedifferentiation‐like transcriptional program, rather than overt cellular senescence.

Pathway enrichment analysis of differentially expressed genes revealed that PS‐NPs‐responsive β‐cells exhibited significant activation of pathways related to pancreatic secretion, antigen processing and presentation, type I diabetes mellitus, cellular senescence, and inflammatory signaling (Figure 3F). When stratified by β‐cell maturation status, immature β cells preferentially enriched pathways associated with stress adaptation and metabolic remodeling, including AMPK signaling, autophagy, toll‐like receptor signaling, and cellular senescence (Figure 3H,I). In contrast, mature β cells displayed enrichment of pathways related to immune regulation, apoptosis, MAPK signaling, and inflammatory responses, indicating distinct transcriptional programs between β‐cell states (Figure 3H,J).

Intersection analysis of enriched pathways between mature and immature β cells identified a shared core of dysregulated processes, including transcriptional misregulation, cellular senescence, and virus‐associated immune pathways, underscoring a prominent immuno‐inflammatory component underlying PS‐NPs‐induced β‐cell identity remodeling (Figure 3K). Collectively, these results demonstrate that PS‐NPs promote a shift in β‐cell state distribution toward immaturity, accompanied by broad transcriptional reprogramming and activation of stress‐ and immune‐related pathways.

### 3.4 PS‐NPs Selectively Reinforce MHC‐I‐Dependent Inflammatory Crosstalk Between DCs and β Cells

**FIGURE 4:**
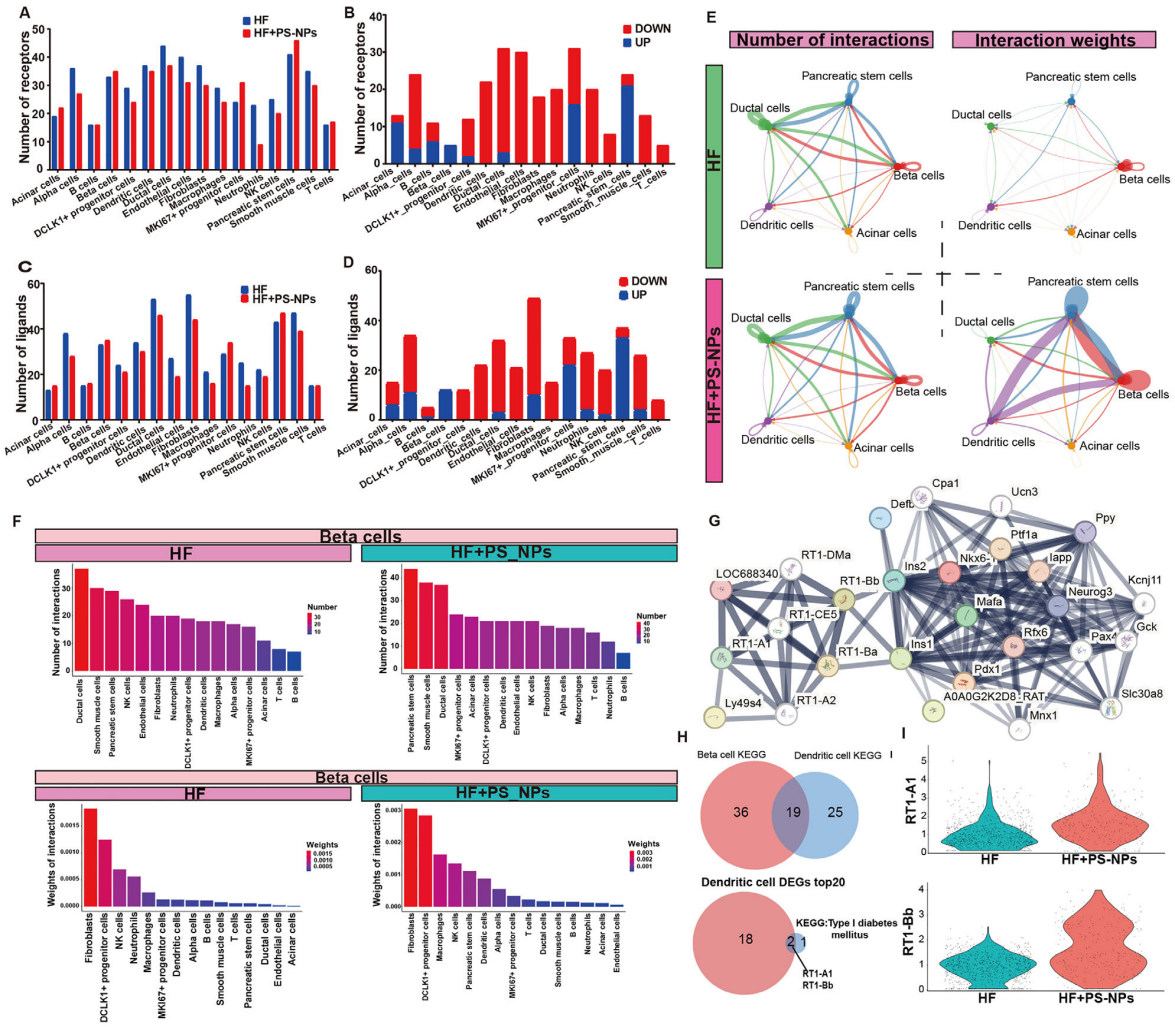
Reshaping of MHC‐I‐dependent DC‐β‐cell communication by PS‐NPs. Cell‐cell communication was inferred using CellChat to quantify ligand–receptor interactions. (A–D) Dot plots comparing the total number of receptors (A, B) and ligands (C, D) between HFD and HFD+PS‐NPs groups. (E) Chord diagram and heatmap showing the strengthened interaction weight specifically between DCs and β cells. (F) Ranking of interaction strength for various ligand‐receptor pairs. (G) Protein‐protein interaction (PPI) network analysis of MHC‐I‐related genes (RT1‐A1, RT1‐Bb). (H) KEGG pathway intersection between DCs and β cells. (I) Violin plots showing the expression levels of RT1‐A1 and RT1‐Bb in the DC population. Abbreviations: DC, dendritic cell; MHC‐I, major histocompatibility complex class I; PPI, protein‐protein interaction; HFD, high‐fat diet. Data information: Interactions were inferred from pooled snRNA‐seq data (*n* = 3/group). Only interactions with *p* < 0.05 were included in the communication analysis.

To delineate how PS‐NPs reshape intercellular communication within the pancreatic microenvironment, ligand‐receptor interaction analysis was performed across major pancreatic cell types. Compared with the HFD group, PS‐NPs exposure resulted in a global reduction in the number of ligand‐receptor interactions across multiple cell populations (Figure 4A–D), indicating an overall attenuation of intercellular connectivity. In contrast, Chord diagram analysis demonstrated that, despite fewer total interactions, β cell‐centered communication networks became more polarized under PS‐NPs exposure. While pancreatic stem cells exhibited the highest overall interaction strength across all groups, the signaling links between β cells and immune‐related cell types, especially DCs, were specifically strengthened following PS‐NPs exposure (Figure 4E). Consistently, quantitative ranking of interaction partners confirmed that while pancreatic stem cells remained a major communication hub, DCs emerged as one of the most significantly enhanced interacting cell types with β cells in the PS‐NPs group, characterized by a substantial increase in both interaction frequency and cumulative interaction weight (Figure 4F).

Network topology analysis identified MHC class I‐related genes, including RT1‐A1 and RT1‐Bb, as central hubs within the β‐cell‐DC interaction network (Figure 4G). Notably, RT1‐A1 and RT1‐Bb were among the top intersecting genes linking DCs DEGs with shared immune pathways, highlighting their potential role as key mediators of immune‐endocrine crosstalk(Figure 4I). Consistent with transcriptomic findings, RT1‐A1 and RT1‐Bb levels were significantly elevated in the HFD+PS‐NPs group compared with the HFD group (Figure 4I). Together, these results demonstrate that PS‐NPs selectively reinforce MHC‐I‐dependent inflammatory signaling between DCs and β cells, suggesting a rewiring of pancreatic cell‐cell communication toward a proinflammatory, diabetes‐relevant network architecture.

### 3.5 PS‐NPs Activate DCs and Upregulate MHC‐I‐Associated Antigen‐Presentation Pathways

**FIGURE 5:**
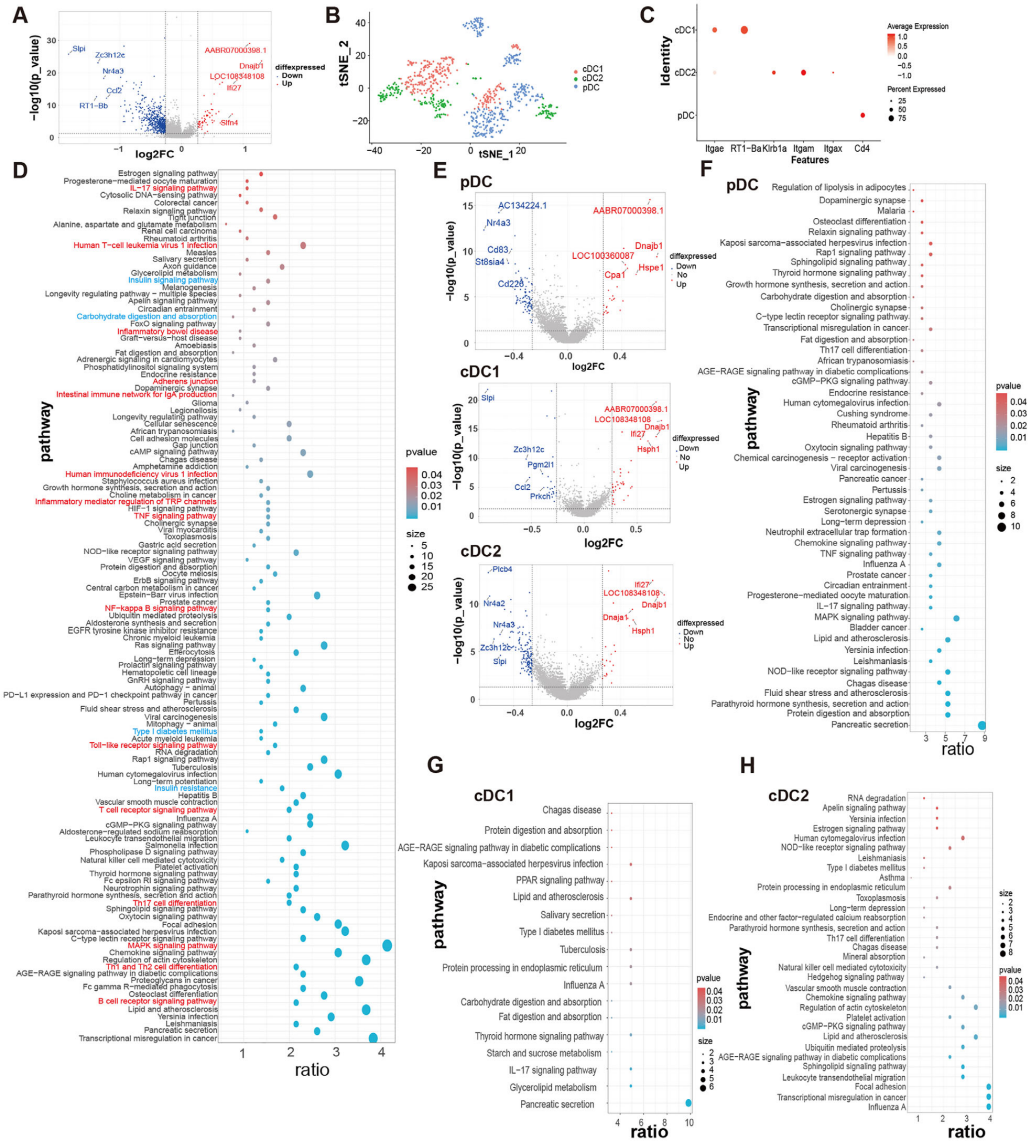
PS‐NPs induce a maturation‐like inflammatory phenotype in DCs. (A) Volcano plot of total DEGs in pancreatic DCs. (B) t‐SNE visualization of DC subclusters (pDC, cDC1, cDC2). (C) Heatmap of canonical markers for DC subset annotation. (D, F–H) KEGG enrichment for total DCs (D) and specific subsets: pDC (F), cDC1 (G), and cDC2 (H). (E) Bar plot showing total DEG counts for each DC subset. Abbreviations: DC, dendritic cell; pDC, plasmacytoid DC; cDC, conventional DC; DEGs, differentially expressed genes. Data information: *n* = 3 biological replicates per group.

PS‐NPs exposure induced widespread transcriptional alterations in pancreatic DCs, with 652 DEGs identified (60 upregulated, 592 downregulated) (Figure 5A). Pathway enrichment revealed prominent activation of inflammatory signaling, together with metabolic reprogramming involving carbohydrate metabolism and pancreas‐associated secretory pathways, as well as type I diabetes‐related pathways (Figure 5B). Clustering analysis further resolved DCs into cDC1, cDC2, and plasmacytoid DC (pDC) subsets (Figure 5C), each of which displayed substantial transcriptional remodeling following PS‐NPs exposure. Notably, cDC2 and pDC subsets showed pronounced enrichment of immune‐activation and antigen‐processing/presentation pathways (Figure 5D).

A consistent signature across all subsets was the robust upregulation of MHC‐I‐related genes, including RT1‐A1 and RT1‐Bb, accompanied by an expanded proportion of pDCs (Figure 5E). These coordinated alterations‐heightened antigen‐presentation machinery, activation‐related transcriptional programs, and pDC expansion‐collectively indicate that PS‐NPs drive a maturation‐like phenotype in pancreatic DCs, associated with enhanced antigen‐presentation potential and inflammatory reprogramming(Figure 5F–H).

### 3.6 MHC‐I Upregulation in DCs Activates TLR4/NF‐κB Signaling in β‐Cell and Drives β‐Cell Dedifferentiation

**FIGURE 6:**
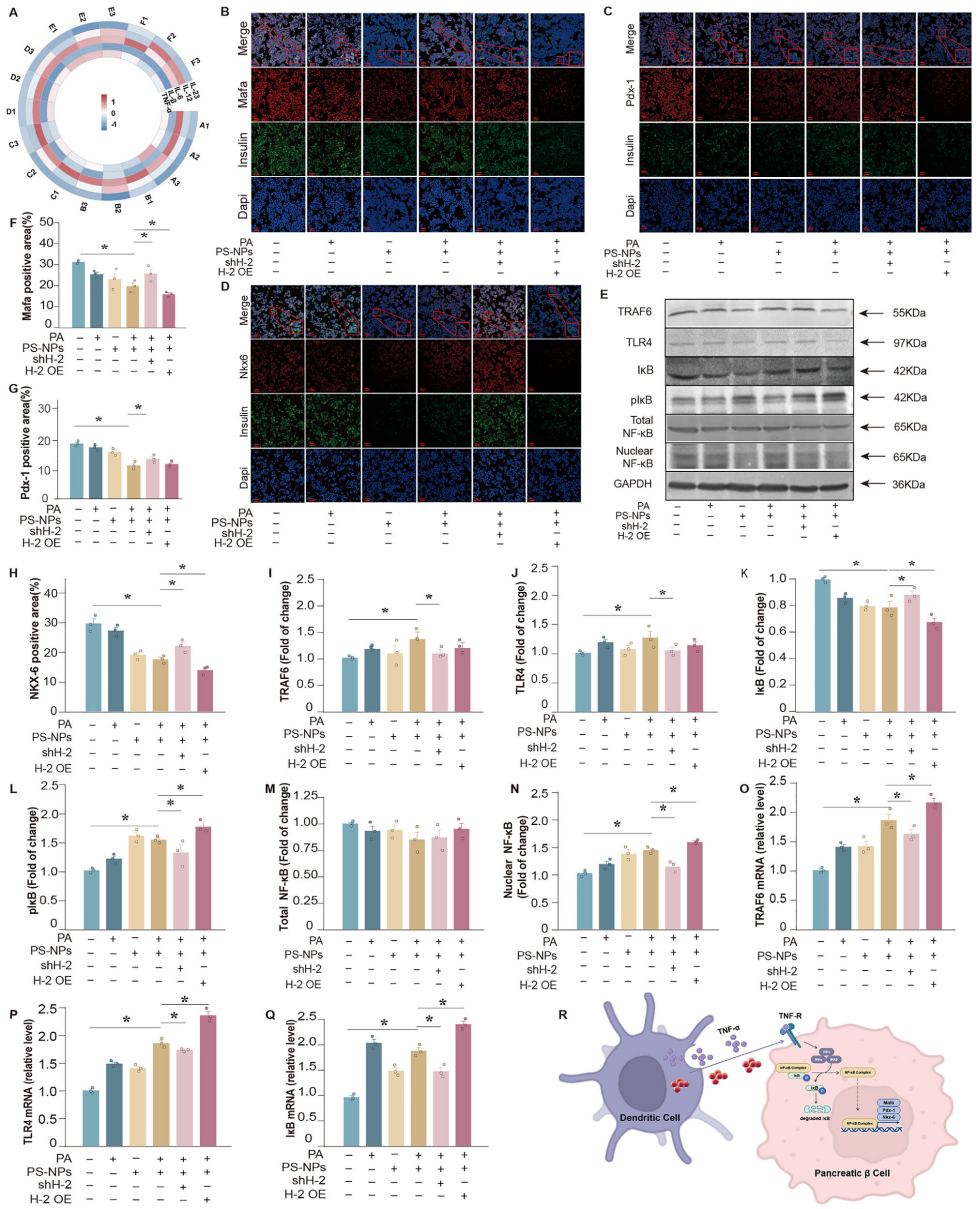
MHC‐I upregulation in DCs drives β‐cell dedifferentiation via TLR4/NF‐κB signaling. Mechanistic validation using an in vitro non‐contact co‐culture system with DC2.4 and 1.1B4 cells. (A) ELISA quantification of proinflammatory cytokines in DC‐conditioned media. (B–D, F–H) mRNA and protein expression of dedifferentiation markers (Mafa, Pdx1, Nkx6.1) in β cells. (E, I–Q) Assessment of TLR4/NF‐κB pathway activation via Western blot (E, I–N) and qPCR (O–Q). (R) Schematic summary of the MHC‐I‐dependent DC‐β‐cell inflammatory axis. Abbreviations: DC, dendritic cell; TLR4, Toll‐like receptor 4; NF‐κB, nuclear factor kappa B; PA, palmitic acid. Data information: All in vitro data represent mean±SEM from *n* = 3 independent biological experiments. Statistical analysis: One‐way ANOVA followed by post‐hoc tests.

To mechanistically validate the role of MHC‐I signaling, we overexpressed H‐2 (the murine homolog of RT1‐A1/RT1‐Bb) in DCs exposed to PA+PS‐NPs. H‐2 overexpression significantly increased inflammatory cytokine secretion (TNF‐α, IL‐1β, IL‐6, IL‐12, IL‐23) (Figure 6A) and markedly increased β‐cell dedifferentiation markers (Mafa, Pdx1, Nkx6.1) in co‐cultured β cells (Figure 6B–D,F–H). These effects were mediated by activation of the TLR4/NF‐κB pathway, evidenced by increased TLR4 and TRAF6 expression, reduced IκB and elevated phosphorylated IκB levels, and enhanced nuclear NF‐κB accumulation (Figure 6E–N, O–Q, Figure S6).

Besides, to determine whether TLR4/NF‐κB signaling is functionally required for β‐cell identity loss, we next employed pharmacological inhibition in the co‐culture system. Pre‐treatment of DCs with the TLR4 inhibitor TAK‐242 or the NF‐κB inhibitor PDTC markedly attenuated PS‐NPs‐induced DC activation and inflammatory cytokine production(Figure S7). Importantly, blockade of TLR4/NF‐κB signaling significantly restored the expression of β‐cell identity markers, including Mafa and Pdx1, demonstrating that activation of this pathway is required for PS‐NPs‐induced β‐cell dysfunction.

We further sought to identify the molecular link between activated DCs and β‐cell TLR4 signaling. Neutralization experiments were performed using MP6‐XT22, a specific TNF‐α‐neutralizing antibody, in DC‐conditioned medium prior to β‐cell treatment. TNF‐α blockade significantly attenuated TLR4 upregulation and downstream NF‐κB activation in β‐cells and effectively rescued the expression of Mafa and Pdx1(Figure S8). These findings indicate that DC‐derived TNF‐α acts as a critical paracrine mediator linking DC activation to β‐cell TLR4/NF‐κB signaling.

Collectively, these results support a mechanistic model in which PS‐NPs‐induced MHC‐I upregulation promotes inflammatory reprogramming of DCs, leading to TNF‐α‐dependent activation of the TLR4/NF‐κB pathway in β cells and ultimately driving β‐cell dedifferentiation (Figure 6R).

### 3.7 Human Occupational Exposure Supports the Role of MHC‐I Dysregulation in Metabolic Impairment

**FIGURE 7:**
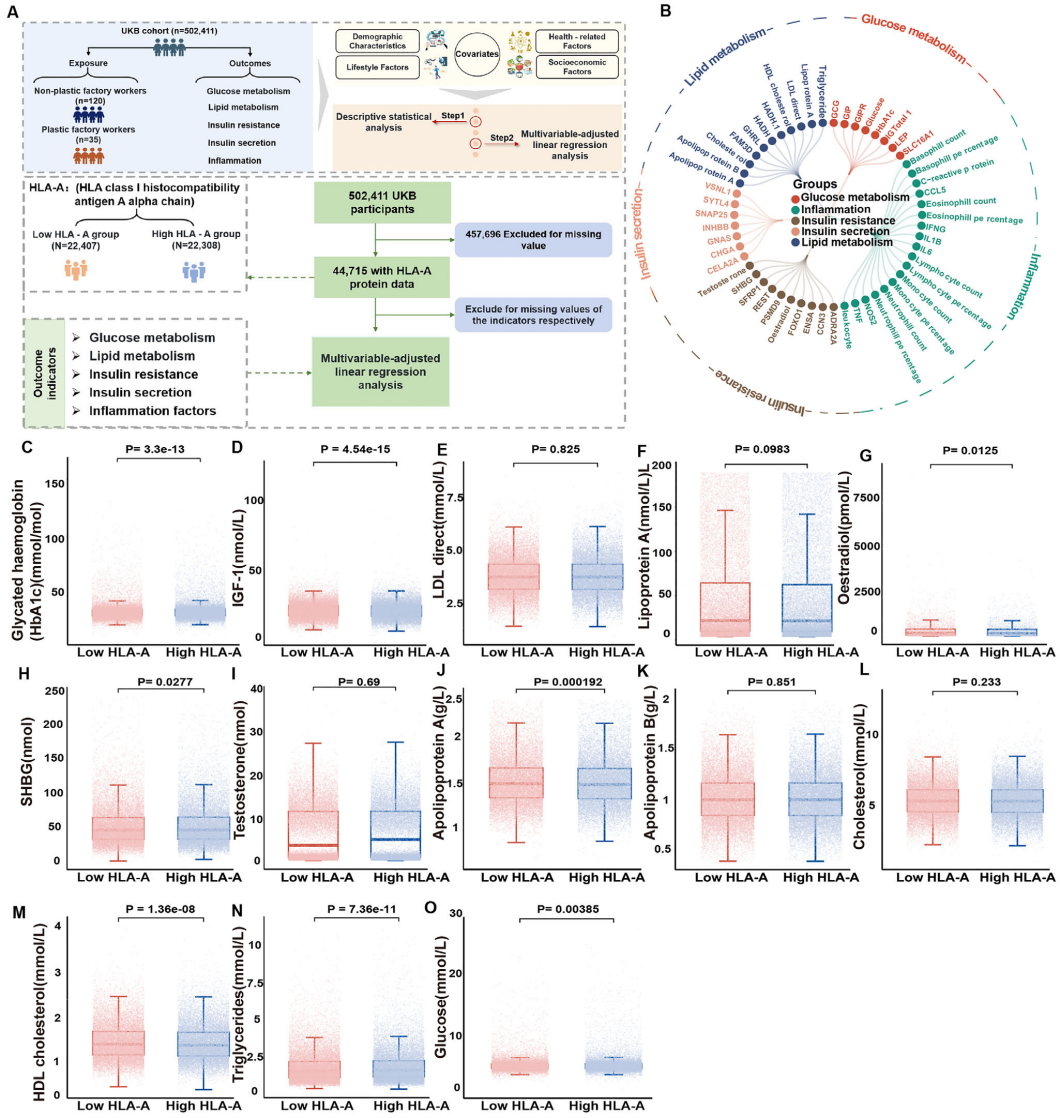
Association between HLA‐A and metabolic impairment in an occupational cohort. (A) Overview of the epidemiological study design using the UK Biobank. (B) Categories of clinical outcomes analyzed. (C–O) Comparison of glucose/lipid metabolism and inflammatory indicators between high and low HLA‐A expression groups. Abbreviations: UKB, UK Biobank; HLA, human leukocyte antigen; BMI, body mass index; HbA1c, glycated hemoglobin. Data information: *n* = 35 participants with occupational plastics exposure and *n* = 120 matched controls.

In a cohort of 155 participants, individuals working in plastics‐related occupations exhibited significantly higher circulating HLA‐A levels compared with controls after adjustment for confounders (β = 0.10, 95% CI: 0.02–0.18; *p* = 0.021) (Figure 7A,B).

Besides, among the participants with lower HLA‐A level, we found the HbA1c(36.07 ± 6.64 mmol/mol vs. 36.52 ± 7.12 mmol/mol), Triglycerides1 (1.71 ± 1.01 mmol/L vs. 1.77 ± 1.04 mmol/L), Glucose (5.12 ± 1.20 mmol/L vs. 5.17 ± 1.33 mmol/L) were significantly increased and IGF‐1(21.47 ± 5.75 nmol/L vs. 21.06 ± 5.72 nmol/L), Oestradiol(463.94 ± 426.06 pmol/L vs. 449.98 ± 390.56 pmol/L), Apolipoprotein A1(1.54 ± 0.27 g/L vs. 1.53 ± 0.27 g/L), HDL cholesterol1(1.46 ± 0.38 mmol/L vs. 1.43 ± 0.39 mmol/L) were significantly decreased compared to the participants with higher HLA‐A (Figure 7C–O), as well as the genes enriched in the pathway of insulin secretion and insulin resistant (Figure S9). To minimize the impact of potential confounding variables, we performed multivariate linear regression analysis. After adjusting for age, sex, race, education level, smoking status, drinking status, and BMI, HLA‐A expression remained significantly associated with fasting glucose (β = 0.015, *p* = 0.002), HDL(β = −0.033, *p* < 0.001), and HbA1c (β = 0.017, *p* < 0.001) (Table S2). These results indicate that the association between the MHC‐I human ortholog and metabolic impairment is independent of common demographic and anthropometric factors. Meanwhile, we observed that the inflammation factors were remarkably increased among the participants with higher HLA‐A levels (Figure S9).

## 4 Discussion

In our study, we provide integrative in vivo, single‐nucleus transcriptomic, in vitro, and human epidemiological evidence that PS‐NPs exposure disrupts pancreatic immune‐endocrine homeostasis through an MHC‐I‐dependent DCs‐β‐cell axis. We demonstrate that PS‐NPs exposure aggravates metabolic dysfunction, promotes β‐cell dedifferentiation, and reshapes the pancreatic immune landscape, with DCs and β cells emerging as the principal PS‐NPs‐responsive populations. Mechanistically, PS‐NPs induce a stress‐activated, maturation‐like phenotype in pancreatic DCs characterized by enhanced MHC‐I expression and inflammatory activation, leading to altered DCs‐β‐cell communication and progressive loss of β‐cell identity. Collectively, these findings support a unified mechanistic framework connecting environmental PS‐NPs exposure to β‐cell dysfunction and metabolic impairment.

Our work provides compelling evidence that the pancreas represents a direct and previously underappreciated target of PS‐NPs toxicity. Prior studies of MPs and NPs have predominantly focused on hepatic, renal, or systemic metabolic consequences, while pancreatic effects have received limited attention, particularly at the level of immune‐endocrine crosstalk. Here, we show that PS‐NPs exposure exacerbates hyperglycemia, impairs glucose tolerance, and induces profound structural and functional alterations within pancreatic islets. Ultrastructural analyses reveal mitochondrial swelling, cristae disorganization, endoplasmic reticulum dilation, and reduced insulin granule density in β cells, collectively indicating substantial organelle stress [16, 17]. Notably, these changes are accompanied by downregulation of key β‐cell identity transcription factors, including Mafa, Pdx1, and Nkx6.1, and by a shift from mature toward immature β‐cell states [18, 19]. This pattern supports β‐cell dedifferentiation as an early and prominent pathogenic response to PS‐NPs exposure, rather than overt cell loss. This distinction is critical, as accumulating evidence indicates that β‐cell identity loss precedes irreversible β‐cell failure and may represent a potentially reversible stage in the progression toward dysglycemia [20, 21].

Single‐nucleus transcriptomic profiling uncovered a complex interactome between endocrine and immune compartments, underscoring the pivotal role of the pancreatic microenvironment in response to PS‐NPs exposure. While bioinformatic analysis also identified pancreatic stem cells (PSCs) as prominent nodes with high interaction weights, our investigation focused on the DC‐β cell axis. This prioritization was driven by the robust and specific upregulation of MHC‐I‐related signaling hubs uniquely localized within the DC population—a molecular signature that directly converges with the observed β‐cell dedifferentiation. Although PSCs are essential for tissue remodeling, the DC‐mediated immune‐endocrine crosstalk appears to be a more proximal driver of the loss of β‐cell identity under PS‐NPs stress.

Although bioinformatic analysis highlighted pancreatic stem/progenitor cell populations as important components of the pancreatic interactome, our data do not support a substantial expansion of bona fide progenitor cells following PS‐NPs exposure. Instead, the observed increase in immature β‐cell states, together with the upregulation of dedifferentiation‐associated markers such as Sox9 and Aldh1a3 and the concomitant loss of mature β‐cell markers, is more consistent with stress‐induced β‐cell identity loss and partial reactivation of developmental programs. This interpretation aligns with emerging evidence that metabolically stressed β cells can transition toward an immature‐like state while largely retaining their lineage identity, rather than undergoing complete conversion into progenitor cells. Therefore, we propose that the immature β‐cell populations identified in our study primarily reflect dedifferentiation‐like remodeling driven by the altered pancreatic microenvironment rather than true progenitor cell expansion. Specifically, PS‐NPs triggered a transcriptional shift in DCs toward a “stress‐activated” state, characterized by enhanced antigen presentation capacity (e.g., RT1‐A1 and RT1‐Bb upregulation). This phenotypic maturation mirrors the DC activation typically elicited by inflammatory or xenobiotic stimuli. Given that nanoparticles are known to activate pattern recognition receptors like TLR4 [22, 23], our findings suggest that PS‐NPs may act as environmental “second hits” that perturb DC homeostasis, thereby reshaping the pancreatic immune milieu and compromising β‐cell stability.

An important finding of this study is the identification of MHC‐I upregulation in DCs as a mechanistic link between PS‐NPs exposure and β‐cell dedifferentiation. Traditionally, aberrant MHC‐I expression has been studied primarily in the context of autoimmune diabetes, where enhanced antigen presentation facilitates CD8^+^ T‐cell‐mediated β‐cell destruction [24, 25, 26]. Emerging evidence, however, suggests that MHC‐I dysregulation may exert broader effects on innate immune signaling, cellular stress responses, and intercellular communication [27, 28, 29]. Our integrative ligand‐receptor analyses and functional experiments support a model in which PS‐NPs‐induced MHC‐I upregulation in DCs amplifies their inflammatory signaling capacity, thereby potentiating cytokine‐mediated activation of stress‐responsive pathways‐including TLR4/NF‐κB signaling‐in neighboring β cells. This inflammatory crosstalk promotes β‐cell identity loss and functional impairment. Importantly, forced overexpression of H‐2 in DCs recapitulated β‐cell dedifferentiation phenotypes, whereas H‐2 silencing partially mitigated these effects, underscoring the causal contribution of this axis. Together, these findings position MHC‐I not merely as a passive antigen‐presenting molecule, but as an active regulator of immune–endocrine communication influencing β‐cell plasticity.

The human data further strengthens the translational relevance of MHC‐I dysregulation in plastic exposure‐associated metabolic dysfunction. Individuals with occupational exposure to plastics exhibited elevated circulating HLA‐A levels alongside metabolic alterations indicative of insulin resistance and impaired glucose handling [30]. Although causality cannot be inferred from this observational analysis, the consistency of MHC‐I upregulation across experimental systems and species supports its potential role as both a biomarker and effector of PS‐NPs‐induced endocrine disruption [31]. These observations raise the possibility that immune remodeling driven by environmental PS‐NPs may precede overt metabolic disease and serve as an early indicator of endocrine vulnerability in exposed populations.

Our findings carry several important implications. First, they identify DCs as key sensors of environmental PS‐NPs exposure and central mediators of pancreatic dysfunction, expanding the scope of nanotoxicology beyond direct cellular injury to encompass immune‐endocrine interactions. Second, they highlight β‐cell dedifferentiation as a critical and potentially reversible mechanism underlying PS‐NPs‐induced metabolic impairment, suggesting that therapeutic strategies aimed at preserving β‐cell identity or modulating DC activation may hold promise. Third, the robust and conserved MHC‐I signature observed across rats, in vitro systems, and humans suggests that MHC‐I‐associated markers could be leveraged for early risk assessment and monitoring of PS‐NPs‐related metabolic disturbances.

Several limitations should be acknowledged. First, our study focused primarily on a high‐fat diet context, which may sensitize pancreatic tissue to environmental insults; whether similar immune‐endocrine mechanisms operate under normal dietary conditions remains to be determined. Second, although our in vitro co‐culture systems recapitulate key aspects of DC activation and β‐cell dedifferentiation, they cannot fully capture the spatial complexity and multicellular interactions of the in vivo pancreatic niche. Third, longitudinal studies are needed to determine whether MHC‐I upregulation represents an initiating trigger or a sustained maladaptive response during chronic PS‐NPs exposure. Fourth, we acknowledge that the absolute differences in metabolic parameters between the HLA‐high and HLA‐low groups in our UK Biobank cohort are relatively small, which is typical for large‐scale population studies of environmental exposures. While these shifts may not reach the threshold for clinical diagnosis of diabetes, they underscore a subtle but persistent metabolic perturbation linked to immune activation. Future longitudinal studies are required to determine whether these minor elevations in glycometabolic markers translate into long‐term disease risk. Finally, a limitation of this study is the relatively small sample size for single‐nucleus RNA sequencing (n = 3 per group), which may not fully capture biological variability among individuals. Some inter‐sample heterogeneity in cell‐type composition was observed; therefore, abundance changes should be interpreted cautiously. However, the major mechanistic conclusions are supported by multiple independent lines of evidence and are unlikely to be substantially affected by this variability.

In conclusion, we delineate a mechanistic framework in which PS‐NPs perturb pancreatic immune‐endocrine homeostasis by inducing MHC‐I‐driven DCs activation and inflammatory crosstalk with β cells, leading to β‐cell dedifferentiation and metabolic dysfunction. As global exposure to PS‐NPs continues to rise, elucidating this immune‐endocrine axis may provide critical insights into early metabolic disease pathogenesis and inform strategies for prevention, biomonitoring, and therapeutic intervention.

## Author Contributions

Wenbo Jiang, Wei Wei, and Tianshu Han conceived the study design. Yuqing Song and Fang Yang completed the animal breeding and molecular biology experiments. Yuqing Song and Xiaoyue Quan did the statistical analysis. Wenbo Jiang and Xinyang Wang wrote the manuscript. Conghui Qiao substantially contributed to additional data analysis, interpretation of results, preparation of responses to reviewers’ comments, and revision of the manuscript. Menghui Guo and Libin Jiang contributed to data verification, literature review, and manuscript revision. Mingyuan Liu provided academic supervision, supported and supervised additional experiments through research funding, contributed to interpretation of the findings, and critically revised the manuscript. All authors reviewed and approved the final manuscript.

## Funding

This work was supported by the China Postdoctoral Natural Science Foundation (2021 M701021 to Wenbo Jiang), the Being High‐Level innovation and Entrepreneurship Talent Support Program leading talent projects (202504841037 to Mingyuan Liu), the Natural Science Foundation of China (82570574), and the Beijing Nova Program (No. 20250484813 to Mingyuan Liu).

## Ethics Approval and Consent to Participate

All the animals experiments were approved by the Medical Ethics Committee of Harbin Medical University and were performed according to the Guide for the Care experiment.

## Conflicts of Interest

The authors declare no conflicts of interest.

## Supporting information




**Supporting File 1**: advs77096‐sup‐0001‐TableS1‐S2.docx.

## Data Availability

The data supporting the findings of our study are available from the corresponding author upon request. The single‐cell RNA sequencing data generated in this study have been deposited in a public repository. The accession information is as follows: Jiang, Wenbo. 2026. “Replication Data for: Polystyrene Nanoplastics Drive beta‐Cell Dedifferentiation through Dendritic Cell‐Intrinsic MHC‐I‐Dependent Inflammatory Crosstalk.” Harvard Dataverse. https://doi.org/10.7910/DVN/2PV6PW.

## References

[1] S. Varma , A. K. Duttaroy , and S. Basak , “Human Exposure to Micro‐ and Nanoplastics: A Mechanistic Perspective of Health Risks Associated with Metabolic and Reproductive Functions,” Science of The Total Environment 989 (2025): 179879, 10.1016/j.scitotenv.2025.179879. 40505506

[2] L. Zhang , B. Wan , J. Zheng , et al., “Polystyrene Nanoplastics Inhibit Beige Fat Function and Exacerbate Metabolic Disorder in High‐Fat Diet‐Fed Mice,” Science of The Total Environment 918 (2024): 170700, 10.1016/j.scitotenv.2024.170700. 38331288

[3] D. Skaba , J. Fiegler‐Rudol , D. Dembicka‐Mączka , and R. Wiench , “Nanoplastics and Immune Disruption: A Systematic Review of Exposure Routes, Mechanisms, and Health Implications,” International Journal of Molecular Sciences 26, no. 11 (2025): 5228, 10.3390/ijms26115228. 40508037 PMC12154010

[4] C. Z. W. Lee and F. M. Spagnoli , “Nurturing Protectors: Macrophages in the Human Pancreatic Islet,” Cell Stem Cell 31, no. 11 (2024): 1553–1554, 10.1016/j.stem.2024.10.006. 39515296 PMC7618580

[5] G. A. Rutter , T. J. Pullen , D. J. Hodson , and A. Martinez‐Sanchez , “Pancreatic β‐Cell Identity, Glucose Sensing and the Control of Insulin Secretion,” Biochemical Journal 466, no. 2 (2015): 203–218, 10.1042/BJ20141384. 25697093

[6] P. P. Khin , J. H. Lee , and H. S. Jun , “A Brief Review of the Mechanisms of β‐Cell Dedifferentiation in Type 2 Diabetes,” Nutrients 13, no. 5 (2021): 1593, 10.3390/nu13051593. 34068827 PMC8151793

[7] M. G. White , J. A. Shaw , and R. Taylor , “Type 2 Diabetes: The Pathologic Basis of Reversible β‐Cell Dysfunction,” Diabetes Care 39, no. 11 (2016): 2080–2088, 10.2337/dc16-0619. 27926891

[8] C. Qian and X. Cao , “Dendritic Cells in the Regulation of Immunity and Inflammation,” Seminars in Immunology 35 (2018): 3–11, 10.1016/j.smim.2017.12.002. 29242034

[9] P. M. Gallo and S. Gallucci , “The Dendritic Cell Response to Classic, Emerging, and Homeostatic Danger Signals Implications for Autoimmunity,” Frontiers in Immunology 4 (2013): 138, 10.3389/fimmu.2013.00138. 23772226 PMC3677085

[10] C. Jiménez‐Cortegana , F. Palomares , G. Alba , et al., “Dendritic Cells: The Yin and Yang in Disease Progression,” Frontiers in Immunology 14 (2024): 1321051, 10.3389/fimmu.2023.1321051. 38239364 PMC10794555

[11] P. P. Nanaware , J. M. Calvo‐Calle , S. D. Redick , et al., “The Antigen Presentation Landscape of Cytokine‐Stressed human Pancreatic Islets,” Cell Reports 44, no. 8 (2025): 115927, 10.1016/j.celrep.2025.115927. 40684438 PMC12624573

[12] M. C. Austin , C. Muralidharan , S. Roy , J. J. Crowder , J. D. Piganelli , and A. K. Linnemann , “Dysfunctional β‐Cell Autophagy Induces β‐Cell Stress and Enhances Islet Immunogenicity,” Frontiers in Immunology 16 (2025): 1504583, 10.3389/fimmu.2025.1504583. 39944686 PMC11814175

[13] Y. Li , F. Sun , T.‐T. Yue , et al., “Revisiting the Antigen‐Presenting Function of β Cells in T1D Pathogenesis,” Frontiers in Immunology 12 (2021): 690783, 10.3389/fimmu.2021.690783. 34335595 PMC8318689

[14] A. Weber , A. Schwiebs , H. Solhaug , et al., “Nanoplastics Affect the Inflammatory Cytokine Release by Primary Human Monocytes and Dendritic Cells,” Environment International 163 (2022): 107173, 10.1016/j.envint.2022.107173. 35303527

[15] L. Xuan , Y. Wang , C. Qu , et al., “Exposure to Polystyrene Nanoplastics Induces Abnormal Activation of Innate Immunity via the cGAS‐STING Pathway,” Ecotoxicology and Environmental Safety 275 (2024): 116255, 10.1016/j.ecoenv.2024.116255. 38552388

[16] H. Lu , V. Koshkin , E. M. Allister , A. V. Gyulkhandanyan , and M. B. Wheeler , “Molecular and Metabolic Evidence for Mitochondrial Defects Associated with β‐Cell Dysfunction in a Mouse Model of Type 2 Diabetes,” Diabetes 59, no. 2 (2010): 448–459, 10.2337/db09-0129. 19903739 PMC2809957

[17] M. Masini , L. Martino , L. Marselli , et al., “Ultrastructural Alterations of Pancreatic Beta Cells in Human Diabetes Mellitus,” Diabetes/Metabolism Research and Reviews 33, no. 6 (2017): e2894, 10.1002/dmrr.2894. 28303682

[18] S. Guo , C. Dai , M. Guo , et al., “Inactivation of Specific β Cell Transcription Factors in Type 2 Diabetes,” Journal of Clinical Investigation 123, no. 8 (2013): 3305–3316, 10.1172/JCI65390. 23863625 PMC3726150

[19] K. L. Webster and R. G. Mirmira , “Beta Cell Dedifferentiation in Type 1 Diabetes: Sacrificing Function for Survival?,” Frontiers in Endocrinology 15 (2024): 1427723, 10.3389/fendo.2024.1427723. 38904049 PMC11187278

[20] K. Amo‐Shiinoki , K. Tanabe , Y. Hoshii , et al., “Islet Cell Dedifferentiation Is a Pathologic Mechanism of Long‐Standing Progression of Type 2 Diabetes,” JCI Insight 6, no. 1 (2021): 143791, 10.1172/jci.insight.143791. 33427207 PMC7821596

[21] N. Brusco , G. Sebastiani , G. Di Giuseppe , et al., “Intra‐Islet Insulin Synthesis Defects Are Associated with Endoplasmic Reticulum Stress and Loss of Beta Cell Identity in Human Diabetes,” Diabetologia 66, no. 2 (2023): 354–366, 10.1007/s00125-022-05814-2. 36280617 PMC9807540

[22] A. E. Zelkoski , Z. Lu , G. Sukumar , et al., “Ionizable Lipid Nanoparticles of mRNA Vaccines Elicit NF‐κB and IRF Responses through Toll‐Like Receptor 4,” NPJ Vaccines 10, no. 1 (2025): 73, 10.1038/s41541-025-01124-x. 40246950 PMC12006303

[23] M. G. Bianchi , M. Chiu , G. Taurino , et al., “The TLR4/NFκB‐Dependent Inflammatory Response Activated by LPS Is Inhibited in Human Macrophages Pre‐Exposed to Amorphous Silica Nanoparticles,” Nanomaterials 12, no. 13 (2022): 2307, 10.3390/nano12132307. 35808143 PMC9268534

[24] K. Yang , Y. Zhang , J. Ding , Z. Li , H. Zhang , and F. Zou , “Autoimmune CD8^+^ T Cells in Type 1 Diabetes: From Single‐Cell RNA Sequencing to T‐Cell Receptor Redirection,” Frontiers in Endocrinology 15 (2024): 1377322, 10.3389/fendo.2024.1377322. 38800484 PMC11116783

[25] E. E. Hamilton‐Williams , S. E. Palmer , B. Charlton , and R. M. Slattery , “Beta Cell MHC Class I Is a Late Requirement for Diabetes,” Proceedings of the National Academy of Sciences 100, no. 11 (2003): 6688–6693, 10.1073/pnas.1131954100. PMC164508 12750472

[26] A. M. Bulek , D. K. Cole , A. Skowera , et al., “Structural Basis for the Killing of human Beta Cells by CD8^+^ T Cells in Type 1 Diabetes,” Nature Immunology 13, no. 3 (2012): 283–289, 10.1038/ni.2206. 22245737 PMC3378510

[27] E. M. Muntjewerff , L. D. Meesters , G. van den Bogaart , and N. H. Revelo , “Reverse Signaling by MHC‐I Molecules in Immune and Non‐Immune Cell Types,” Frontiers in Immunology 11 (2020): 605958, 10.3389/fimmu.2020.605958. 33384693 PMC7770133

[28] S. Liu , T. Jayasinghe , D. Kaminska , et al., “Augmented MHC Class I on Professional Antigen‐Presenting Cells and Enhanced Cytokine Production by CD8^+^ T Cells in Type 2 Diabetes Mellitus,” European Journal of Immunology 55, no. 12 (2025): 70109, 10.1002/eji.70109. PMC12716204 41416940

[29] D. P. Granados , P.‐L. Tanguay , M.‐P. Hardy , et al., “ER Stress Affects Processing of MHC Class I‐Associated Peptides,” BMC Immunology 10, no. 1 (2009): 10, 10.1186/1471-2172-10-10. 19220912 PMC2657905

[30] F. Mahmud , D. B. Sarker , J. A. Jocelyn , and Q. A. Sang , “Molecular and Cellular Effects of Microplastics and Nanoplastics: Focus on Inflammation and Senescence,” Cells 13, no. 21 (2024): 1788, 10.3390/cells13211788. 39513895 PMC11545702

[31] H. J. Tyc , K. Kłodnicka , B. Teresińska , R. Karpiński , J. Flieger , and J. Baj , “Micro‐ and Nanoplastics as Disruptors of the Endocrine System—A Review of the Threats and Consequences Associated with Plastic Exposure,” International Journal of Molecular Sciences 26, no. 13 (2025): 6156. 40649932 10.3390/ijms26136156 PMC12249724

