# Supplementary material for: Polystyrene Nanoplastics Drive β‐Cell Dedifferentiation Through Dendritic Cell‐Intrinsic MHC‐I‐Dependent Inflammatory Crosstalk
Source: Adv Sci (Weinh). 2026 Aug 17:e77096. Online ahead of print. doi: 10.1002/advs.77096 (PMC13479589; doi:10.1002/advs.77096)
Supplement: Supplementary file 1 [file ADVS-9999-e77096-s001.docx]

**S-Table1. Baseline characteristic of the participants based on the HLA-A protein level.**

| **Variable** | **Overall**  N = 44,715^1^ | **Lower HLA-A** N = 22,407^1^ | **Higher HLA-A**  N = 22,308^1^ | **p-value**^2^ |
| --- | --- | --- | --- | --- |
| **Age** (years) | 56.82 (8.19) | 56.32 (8.24) | 57.33 (8.10) | <0.001 |
| **Sex** (Female) | 24,190 (54.1%) | 12,184 (54.4%) | 12,006 (53.8%) | 0.2 |
| **Race** (White) | 36,788 (82.3%) | 18,586 (82.9%) | 18,202 (81.6%) | <0.001 |
| **BMI** (kg/m²) | 27.46 (4.81) | 27.37 (4.73) | 27.55 (4.89) | <0.001 |
| **TDI** | -1.16 (3.20) | -1.18 (3.19) | -1.14 (3.20) | 0.133 |
| **Education** (High school or above) | 24,955 (55.8%) | 12,674 (56.6%) | 12,281 (55.1%) | 0.001 |
| **Smoking status** (Current) | 4,785 (10.7%) | 2,367 (10.6%) | 2,418 (10.8%) | 0.351 |
| **Alcohol drinker status** (Current) | 40,724 (91.1%) | 20,558 (91.7%) | 20,166 (90.4%) | <0.001 |
| **MET**(min/week) | 2,629.88 (2,687.65) | 2,659.69 (2,693.50) | 2,599.83 (2,681.48) | 0.003 |

^1^Mean (SD); n (%)

^2^Wilcoxon rank sum test; Pearson's Chi-squared test

S-**Table2. Association between HLA-A expression and glycolipid metabolic indicators in multivariate linear regression after confounders adjustment‌**

|  | **N** | **β** | **95%CI**  **(Lower Bound)** | **95%CI**  **(Upper Bound)** | **P** |
| --- | --- | --- | --- | --- | --- |
| IGF-1 | 42,284 | -0.027 | -0.604 | -0.294 | <0.001 |
| HbA1c | 42,316 | 0.017 | 0.155 | 0.502 | <0.001 |
| LDL | 42,409 | -0.006 | -0.041 | 0.008 | 0.185 |
| Lipoprotein A | 34,009 | -0.009 | -2.901 | 0.205 | 0.089 |
| Oestradiol | 6,808 | -0.008 | -35.839 | 17.854 | 0.511 |
| SHBG | 38,568 | 0.023 | 1.187 | 2.612 | <0.001 |
| Apolipoprotein B | 42,255 | -0.007 | -0.012 | 0.002 | 0.171 |
| Cholesterol | 42,505 | -0.014 | -0.079 | -0.014 | 0.004 |
| HDL cholesterol | 38,905 | -0.033 | -0.047 | -0.028 | <0.001 |
| Triglycerides | 42,475 | 0.015 | 0.019 | 0.074 | 0.001 |
| Glucose | 38,883 | 0.015 | 0.02 | 0.093 | 0.002 |
| Testosterone | 38,365 | 0.001 | -0.056 | 0.101 | 0.579 |
| Apolipoprotein A | 38,680 | -0.013 | -0.017 | -0.002 | 0.009 |

Adjustment with age, sex, race, education level, smoking status, drinking status and BMI.

LDL: ‌Low-Density Lipoprotein‌; HDL: High-Density Lipoprotein; SHBG: Sex Hormone Binding Globulin; ‌
